# Case of Acute Rheumatism of the Stomach, with Remarks on Purgatives, &c.

**Published:** 1820-02

**Authors:** C. W. Smerdon

**Affiliations:** Member of the Royal College of Surgeons.


FOR THE LONDON MEDICAL AND PHYSICAL JOURNAL.

### *Case of Acute Rheumatism of the Stomach, with Remarks on Purgatives, &c.* By C. W. Smerdon, Esq. Member of the Royal College of Surgeons.

MAY 15, 1818.—Miss E. B. aged 18, delicate constitution, is attacked with acute rheumatism, principally in the ankles and wrists: had an attack of the same kind last year,—pyrexia, furred tongue, great thirst, bowels constipated. To take a cathartic senna draught, and apply a tight bandage to each ankle.

*Evening*.—The draught has operated severely; was requested to see her immediately, in consequence of excruciating pain of the stomach, which comes on in paroxysms, after very short intervals. During their continuance her screams are truly alarming, and she is obliged to be held in bed. No sickness; pressure on the epigastrium so acutely painful, that the weight of the bed-cloths is distressing to her; pulse small and feeble, at 120; great prostration of strength. Castor-oil, with a few drops of laudanum, relieved her for an hour, when the paroxysms returned as severe as before. V. S. ad lbj. blood flowed slowly, in consequence of exhaustion; fainted. Fomentations to the bowels, and magnesia at intervals.

*May* 16*th*.—Early this morning pain returned with increased violence; countenance expressive of great horror; extreme exhaustion, particularly between the paroxysms; bowels not been opened by the oil; pulse very feeble and thready; thirst; extremities cold. V.S. ad ℥ xx. Blood flowed more freely; fainted; no vomiting. Hirudines xv. epigastrio; Enemata purgant. com.

*Evening*.—Has been tolerably easy with respect to acute paroxysms; but the slightest pressure on the epigastrium, and even motion of the body, occasion great pain. Blood buffy; pulse very quick and thready. Leeches bled very freely; no sickness. Injections have brought away but little fæces. Rep. Ol. Ricini.

17*th*.—Has had no severe paroxysms since yesterday morning; but pain, on pressure over the stomach, still exquisite. Tongue furred; scanty evacuations; countenance depressed; pulse very quick and feeble; ankles and wrists very painful. Capiat hodie magnesiæ gr. xx. quartis horis; et Extr. Colocy. comp. gr. vi., calomel, gr. vi. hora somni.

18*th*.—Has had a restless night; pain, on pressure over the stomach, not abated. Bowels have been scantily opened; fæces fetid and pitchy, in other respects the same as yesterday. Rep. Oleum Ricini statim. In the evening, the paroxysms of the stomach returned with increased severity, and which are so exquisite, that her screams have alarmed the neighbours. After remaining in this state for about an hour, the disease was suddenly translated to the muscles of the larynx and contiguous parts. The throat feels hard, and is much swollen; respiration laborious; the tongue is thrust forward, and she cannot articulate.

V.S. ad lbj.

Bleeding has given her great relief, but she is left in a state of great exhaustion.

℞ Hydrarg. submur.
Pulv. Antimon. ana gr. v.
Extr. Colocynth. comp. gr. x. M. f. Pilulæ vj. statim sumendæ.

19*th.*—Has had a restless night, but tolerably free from pain; bowels have been more freely opened; fæces extremely black; and has evacuated per anum a large round worm. Blood buffy, and cupped; pain, on pressure over the stomach, very great. In the evening, the rheumatic paroxysms came on with equal severity as last night, first in the extremities, then in the stomach and muscles of the tongue and throat, but were somewhat less durable: mind has wandered much this evening; extreme exhaustion on the subsidence of the paroxysms. To-day she has taken a senna mixture with salts, every four hours, which has produced several black and fetid stools. To take the calomel pills at bed-time.

24*th.*—Since last report she has regularly taken the aperient mixture every four hours, and the pills every alternate night, with evident advantage. The rheumatic paroxysms have gradually left the stomach and throat, but remain unabated in the ankles and wrists. The evacuation by the bowels have not improved in colour, and fever regularly comes on at night at the usual hour: as she is tired of medicine, pills of blue pill and cathartic extract are ordered every night, and an embrocation for the painful extremities.

26*th.*—Her bowels have been confined since last report, and, consequently, she has not been so well. Her fever has likewise increased: she has had transient paroxysms of pain of the stomach, whilst that of the extremities has wholly deprived her of rest. Capt. haust. sennæ cum potassæ tart. ℥vj. statim.

27*th.*—Is in less pain to day: bowels relieved; fæces black. Rep. haust. aperiens.

28*th.*—Bowels have been freely evacuated; has, consequently, had less pain; complains of great exhaustion; and is very anxious to leave off the purgatives. Appetite returning.

Cap. Decoct. Cinchonæ cum soda, ter die.

℞ Pilulæ Calomel. comp. gr. vj.

——— Cambogiæ comp. gr. vii. M. f. Pilulæ ij. omni nocte sumendæ.

30*th.*—The same. Bowels have been scantily evacuated. Wandering pains of the extremities still very distressing.

Rep. Mistura cinchon.

℞ Pil. Calomel. comp. gr. vj.

Extr. Colocy. comp. gr. vii. M. f. Pilulæ ij. omni nocte sumendæ.

*June* 1*st.*—The bark apparently increases the nocturnal pain and fever, and therefore to be omitted. Capt. omni nocte, cum pilulis, pulv. antim. gr. v. This plan was persisted in with the best effect until the 5th, when the nocturnal pains were so far weakened, and her general health so much improved, that medical attendance was no longer deemed necessary.

Here is a case of acute rheumatism attacking the extremities of a delicate young female, in whom the functions of the chylopoietic viscera were faulty. The stomach, being thus rendered highly susceptible to the impression of whatever disease might be going on in another part of the body, is also attacked with rheumatism by metastasis from the extremities; but which, it is presumed, would not have happened had the organ been previously in a healthy state. The sceptic, who ridicules the modern theory on disordered digestive function, and who denies the probability of its being the cause of innumerable morbid sympathies, only because the chain which connects them is hid from his view, will consider the opinion which I have just given entirely gratuitous. He may ask what proof can be drawn from the statement of the case for such an assumption? I answer,—the apparent good derived from a long course of drastic purges; the continued black colour of the fæcal evacuations; and, particularly, the worm voided per anum. Hence, during the fevers of different subjects, in one the brain will become affected with the inflammation, in another the stomach, in another the liver. Now, we are not to infer from hence that there are various proximate causes of fever, or that that cause is inflammation; but that each affected organ, previous to the febrile attack, was in a weakened state, and therefore rendered unable to resist the stimulus arising from a suddenly increased circulation.

Rheumatic inflammation of the internal organs is, I believe, a very rare disease; probably because they are not exposed to the vicissitudes of climate: that the case just narrated is one of acute rheumatism of the stomach cannot, I think, be doubted; and likewise that its cure was effected, not by the common remedies for rheumatism, but by a persevering continuance of drastic purges.

The anatomical structure of the intestines shews that two sorts of liquors are poured into them, which doubtless have not only their separate uses in the animal economy, but also their excess or diminution must be the sources of derangements of the general health. The glandular secretions being the products of more complicated structures than the liquors which are derived from mucous or serous membranes, are consequently more easily deranged, and, when deranged, are more difficultly cured. Practice also points out to us that, in general, very different sorts of intestinal evacuations are the products of saline and drastic or mercurial purges; and that in most cases where the chylopoietic viscera are the sources of general derangement, as also in those where the secretions of the viscera are in an inactive state, (for instance, in idiopathic fever,) infinitely more good may be derived from the use of the latter than from that of the former, notwithstanding the products of each, with respect to quantity, shall be the same. Hence then it appears to me evident, that the more complicated structure of the secretory glands of the chylopoietic viscera (in which the liver may be included) requires, when their action is sluggish, or morbid, more powerful excitants than the simpler structure of the mucous membrane; the remedy for the one being the drastic purges, and for the other the saline.

It must be acknowledged, I presume, that more diseases are curable through the medium of purgatives acting upon the bowels, than by any other means whatsoever: hence the liver being a complicated organ, the physiology of which is but imperfectly known, has been considered the great cause of all those morbid affections of that general system which the action of purgatives is capable of relieving. This sweeping conclusion is not only inimicable to the improvement of medical knowledge, but is also become so generally adopted by the public, (at least of this country) that with them bile and disease are synonimous terms. Easily deranged as the functions of the liver certainly are, (and which, from the acknowledged importance of the viscus, must lead to derangements of the general health,) there are other organs belonging to the same system, of equal importance and of equal delicacy. The large intestines, from their situation, great extent of surface, and the numerous glands which pour their liquors into them, are, I am convinced, well deserving the attention of the medical philologist.* Here the rejected matters from the upper bowels undergo an entire change; they lose their bitter taste; become feculent; and lastly, acquire consistency, and peculiarity of colour and shape. All these facts, however, would be of no consequence, if experience and observation had not proved, that a deviation from the healthy standard of any of these appearances of the evacuated matters, is always attended by a derangement of the general health.

The bile is considered to be the sole cause of colour in the fæcal evacuations; but this opinion is grounded on a very feeble foundation; and, indeed, an enlarged view of the subject, unprejudiced, must shew, that, although it be a principle colouring ingredient, it is not the only one. The bilious secretion of all animals is, I believe, chemically the same; consequently the

* I am well aware that many diseases are curable by a well-directed course of purgatives, the origin of which cannot be ascribed to the derangement of any one particular organ, but to a morbid action either of the sanguiferous or nervous system: the curative process in such cases is not very obscure; but, in order to satisfy, or blind the inquisitive, it is by no means unfrequent to hear medical men talk about bile sticking to the kidneys, stomach, intestines, and even the lungs:—such language is truly ridiculous, and is deserving of the severest reprehension.

fæcal colour of them all should be similar: but this is so far from being the case, that the intestinal excretion of every animal is peculiar as well in colour as in figure and smell. Here, then, we have direct proof that something else must act upon the rejected matters to make them feculent, and this can nowhere be obtained but in the large intestines.

In mice, which have neither gall-bladder nor colon valve, the bile is intensely bitter, limpid, and almost colourless. When the stomach of this animal is distended with food recently received, the two first intestines, particularly the duodenum, have a very vascular appearance, and contain a quantity of bitter mucus. When this food has undergone the necessary change, and a portion of it has passed out of the stomach, the superior part of the alimentary canal contains a bland cream-coloured substance, here and there streaked with yellow, and very bitter. This appearance of the chylous matter continues as far as the lower part of the ileum, when it suddenly becomes very dark,—a colour which rather increases as we approach the extremity of the tube, whilst the fæces (as they now may be called) feel gritty, and to the taste have almost wholly lost the bitter principle of the bile. In the rectum they become hardened, and receive the form which we see when evacuated per anum.

These observations, I presume, fairly warrant the conclusion, that, as chylification goes on in the small intestines, so another process is performed in the large, between the glandular and mucous secretion of the intestines, the rejected chylous matter, and the bile; and that this process alone gives colour and other peculiarities to the excretum of the bowels.

*Clifton, Bristol; Dec.* 1819.

